# Specific features of human monocytes activation by monophosphoryl lipid A

**DOI:** 10.1038/s41598-018-25367-y

**Published:** 2018-05-04

**Authors:** Ryme Chentouh, Catherine Fitting, Jean-Marc Cavaillon

**Affiliations:** Unit “Cytokines & Inflammation”, Institut Pasteur, Paris, France

## Abstract

We deciphered the mechanisms of production of pro- and anti-inflammatory cytokines by adherent human blood mononuclear cells (PBMC) activated by lipopolysaccharide (LPS) or monophosphoryl lipid A (MPLA). Both LPS and MPLA induced tumor necrosis factor (TNF) production proved to be dependent on the production of interleukin-1β (IL-1β). Of note, MPLA induced IL-1β release in human adherent PBMCs whereas MPLA was previously reported to not induce this cytokine in murine cells. Both LPS and MPLA stimulatory effects were inhibited by Toll-like receptor-4 (TLR4) antagonists. Only monocytes activation by LPS was dependent on CD14. Other differences were noticed between LPS and MPLA. Among the different donors, a strong correlation existed in terms of the levels of TNF induced by different LPSs. In contrast, there was no correlation between the TNF productions induced by LPS and those induced by MPLA. However, there was a strong correlation when IL-6 production was analyzed. Blocking actin polymerization and internalization of the agonists inhibited MPLA induced TNF production while the effect on LPS induced TNF production depended on the donors (i.e. high TNF producers *versus* low TNF producers). Finally, conventional LPS, tolerized adherent PBMCs to TLR2 agonists, while MPLA primed cells to further challenge with TLR2 agonists.

## Introduction

Endotoxins (lipopolysaccharide, LPS) are among the most potent bacterial activators of immune cells. As a consequence, LPSs display numerous favorable bioactivities including anti-tumor activity, pyrogenicity, and radioprotection. However, LPSs also have deleterious bioactivities such as: capillary leak, coagulation, tissue toxicity, and lethality. The variety in responses to LPSs is reflected in the biochemical diversities of endotoxins^1^. One specific feature of endotoxins is their strong capacity to induce cytokine release. Their capacity to induce interleukin-1 (IL-1) was first reported in 1972^2^, and endotoxins contributed to the discovery of tumor necrosis factor (TNF)^3^. TNF was later shown to contribute to the toxic effects of LPSs^4^. IL-1 and TNF orchestrate the inflammatory and innate immune response within auto-amplificatory loops^5,6^. The lipidic moiety, called lipid A, is recognized as the active part of the molecule^7^. It is important to note that the keto-deoxyoctulosonate (KDO), the sugar that links the lipid A to the polysaccharide moiety, also contributes to the bioactivity of LPS^8^. Lipid As have a common backbone consisting of a β-1,6-linked D-glucosamine disaccharide carrying ester and amide-linked fatty acids, and contain phosphate groups at positions C-1 and C-4′. Phosphate groups can be substituted by L-arabinosamine or phosphoethanolamine.

Among the numerous bioactivities of endotoxins, adjuvanticity was reported in 1956^9^. In the quest of identifying new adjuvants, tremendous efforts have been made to dissociate the beneficial properties of LPS from the toxic ones. These efforts resulted in the discovery of the monophosphoryl lipid A (MPLA). MPLA is an analog of the lipid A moiety^10^ and was approved on October 2009 by the US Food and Drug Administration as a new adjuvant. This adjuvant is currently used in vaccines against melanoma, human papilloma virus, and hepatitis B.

LPSs activate monocytes/macrophages after binding to the cell’s CD14, which shuttles the molecule to the Toll-like receptor 4 (TLR4) + Myeloid differentiation protein 2 (MD2) complex. The bound complex leads to the activation of two signaling cascades, one depending on the adaptor proteins myeloid differentiation factor 88 (MyD88), and the other on Toll-interleukin 1 receptor domain-containing adapter inducing interferon-beta (TRIF)^11^. More recently, the intracellular interaction of LPS with murine caspase 11 or human caspase 4 & 5 was identified as another activation pathway^12^. CD300b has also been shown to been engaged in the LPS-induced cytokine response^13^. In mice, the low toxicity of MPLA has been associated with the bias toward the TRIF signaling pathway^14^.

It is important to keep in mind that most knowledge on these pathways has been acquired using murine cells. The use of murine cells may represent a limitation of our current understanding of the events occurring in humans. Mice are known to be extremely resistant to endotoxin while humans are 10^5^ times more sensitive^15^. A reported self-injection of LPS (28 ng/kg in a healthy individual) resulted in admission into the intensive care unit for appropriate care^16^. Furthermore, another reported self-injection with a higher dosage (15 µg/kg) caused shock and multiple organ failure^17^. These reports along with the known murine lethal dosage of greater than 25 mg/kg underline the vast difference of sensitivity between the two species. Most importantly, among lipid A analogs and lipid A precursors some can behave as agonists in murine macrophages while acting as antagonists in human macrophages^18,19^. Accordingly, we have further deciphered the activation of human monocytes upon exposure to MPLA, compared to LPS. Using human monocytes, we have established that MPLA display numerous differences when compared to LPS in its capacity to induce cytokine production in humans and we reveal some key differences to the knowledge that was acquired with the use of murine cells.

## Material and Methods

### Reagents

All TLR ligands and inhibitors were reconstituted and stored following the manufacturer’s instructions. Conventional lipopolysaccharide (cLPS) from *Escherichia coli* O111:B4 (Sigma-Aldrich), highly purified lipopolysaccharides from *Salmonella abortus equi* S-form (hpLPS) (Enzo Life science), *Salmonella minnesota* R595 (ReLPS) (Enzo Life science), synthetic TLR2 agonists Pam_3_CysSK_4_ (EMC microcollections), and Pam_2_CysSK_4_ (EMC microcollections) were used at a concentration of 100 ng/mL. Synthetic monophosphoryl lipid A from *Escherichia coli* (MPLA), the TLR7/8 agonist R848, the TLR3 agonist Poly I:C, and the dectin-1 agonist Zymosan depleted (zymosan) were all purchased from Invivogen. MPLA and R848 were used at a concentration of 1 µg/mL, Poly I:C was used at a concentration of 50 µg/mL, and zymosan was used at a concentration of 100 µg/mL. The TLR4 inhibitors, LPS from *Rhodobacter sphaeroides* (Invivogen) and Eritoran (a kind gift of Dr. Lynn Hawkins, Eisai, Co., Ltd.) were used at a concentration of 1 µg/mL or 10 µg/mL, and 10 nM or 100 nM respectively. The actin polymerization inhibitors, cytochalasin D and latrunculin A, were both purchased from Tocris and used at a concentration of 3 µM and 2 µM respectively. The pan caspase inhibitor (Z-VAD-FMK) was purchased from Invivogen and used at a concentration of 20 µM. The IL-1R antagonist (IL-1Ra) Anakinra was a kind gift from Dr. Oumaïma Granet (Pasteur Institute, Paris), it was used at a concentration of 100 µg/mL. The Syk inhibitor (R406) was purchased from Invivogen and used at a concentration of 1 or 5 µM. The human anti-CD14 antibody (hCD14Ab) was a monoclonal mouse IgG1 from R&D systems and was used at a concentration of 2.5 µM.

### Monocytes isolation

Blood from thirty-four healthy donors was collected after apheresis by the French blood bank (EFS, Établissement français du sang) on the same day as the assays were performed. The donors had consented that their blood will be used for research. All methods and all experimental protocols were carried out in accordance with relevant guidelines and regulations of Institut Pasteur. The blood was diluted 1:2 in RPMI-1640 medium (Lonza) and peripheral blood mononuclear cells (PBMC) were isolated after the diluted blood was layered on the top of a Ficoll-Hypaque solution (lymphocyte separation medium, Eurobio). The cells were washed twice in RPMI-1640. The concentration and the cell viability were assessed on a Malassez cell after eosin staining. The suspension was adjusted to 6 × 10^6^ PBMC/mL with RPMI-1640. PBMCs were plated in 48-wells plates, 250 µL/well. Monocyte enrichment was performed by adherence for two hours at 37 °C in a humidified atmosphere of 95% oxygen and 5% carbon dioxide. Non-adherent cells were removed by flipping-over the plates and the plates were washed twice before further use.

### Cell culture

After adherence, fresh culture medium was added. Culture medium consisted of RPMI-1640, antibiotics (penicillin 100 U/mL; streptomycin 100 µg/mL) and 0.2% heat-inactivated human serum (Biowhittaker). Cells were then incubated with TLR ligands for twenty hours at 37 °C in a humidified atmosphere. At hour twenty, supernatants were harvested and stored at −20 °C before further analysis.

### Inhibition assays

When required, alternative antagonists were used before TLR ligand exposure. The Syk inhibitor, R406, required a one-hour incubation period, while the pan-caspase-inhibitor, and the endocytosis inhibitors required an incubation time of thirty-minutes. The TLR4 inhibitors, as well as, the hCD14Ab and the IL-1Ra did not need an incubation period and were added just prior to the TLR ligand stimulation. Cytochalasin D, latrunculin, pan-caspase inhibitor, or Anakinra were present during the entire incubation period. Cytochalasin D, and latrunculin were added thirty minutes before the addition of the agonists. For an easier visualization of the effects of each of the individual drugs, the results of each individual donor is provided as a percent of the cytokine production in the presence of the drug, where 100% is attributed to the cytokine value in the absence of the drug. The calculation is based on the following formula: [cytokine (ng/mL) in the presence of the drug]/[cytokine (ng/mL) in the absence of the drug] × 100.

### Tolerance assays

The ligands were used at the same concentration during the pretreatment phase and the challenge phase for the tolerance experiments. After retrieving supernatants at hour twenty, cells were washed twice with RPMI-1640, and fresh culture medium was added. The cells were then challenged with TLR ligands for another twenty hours. At hour forty, the supernatants were collected and stored at −20 °C before further analysis.

### Cytokine measurements

Cytokine production was assessed at hour twenty for TNF, IL-1β, IL-10, IL-1Ra, and IL-6 using specific ELISA duoset kits from R&D systems according to the manufacturer’s instructions. Cytokine production was also assessed for the tolerance experiments at twenty hours after challenge for both TNF and IL-1β using the same methodology.

### Western blots

In order to perform the Western blots, cells were cultured on 6-wells plates. At different time points after stimulation, 15 minutes, 30 minutes, and 60 minutes adherent monocytes were washed using PBS (Lonza) and stored at −80 °C before being processed for protein extraction. Proteins were extracted using RIFA buffer (Sigma Aldrich), lysis reagent, and mechanical scraping. Protein extracts were then quantified using a Pierce BCA assay kit (Thermofischer scientific). After quantification, protein extract samples were diluted and reduced by boiling for 5 minutes in 4x Laemmli Sample buffer (Bio-Rad) supplemented with β-mercaptoethanol. The samples (25 µg per lane) were then separated on 10% polyacrylamide Mini-PROTEAN TGX Stain-Free^TM^ Precast gels (Bio-Rad) and transferred to a nitrocellulose membrane using the Trans-blot turbo transfer system (Bio-Rad). Membranes were blocked with 5% milk blocking buffer for one hour at room temperature. Membranes were incubated with primary antibody overnight at 4 °C on a rocker according to the manufacturer’s instructions. The following antibodies were purchased from Cell Signaling Technology: p38 MAPK antibody, phospho-p38 MAPK (Thr180/Tyr182) (D3F9) XP rabbit mAb, p44/42 MAPK (Erk1/2) antibody, phospho-p44/42 MAPK (ERK1/2) (Thr202/Tyr204) antibody, IκBα (44D4) rabbit mAb, γ-Tubulin antibody, SAPK/JNK antibody, phospho-SAPK/JNK (Thr183/Tyr185) (81E11) rabbit mAb, Syk (D3Z1E) XP rabbit mAb, and phosphor-Syk (Tyr525/526) (C87C1) rabbit mAb. Afterwards, membranes were washed and incubated with a secondary antibody from Jackson Immunoresearch. Visualization was performed using Clarity Western ECL blotting substrates from Bio-Rad and analysis was done on an Amersham imager 600 (GE healthcare).

### Statistical analysis

Data were expressed as median with interquartile range. Comparisons of different groups were done via Kruskal-Willis test with Dunn’s correction for data that did not pass D’Agostino & Pearson normality test or a one-way ANOVA with Dunnett correction for data that did pass D’Agostino & Pearson normality test. All analyses were done using GraphPad Prism 7 statistical software (GraphPad Software). A p value of < 0.05 was considered as statistically significant.

## Results

### TNF and IL-1β production: high and low responders

**Figure 1:**
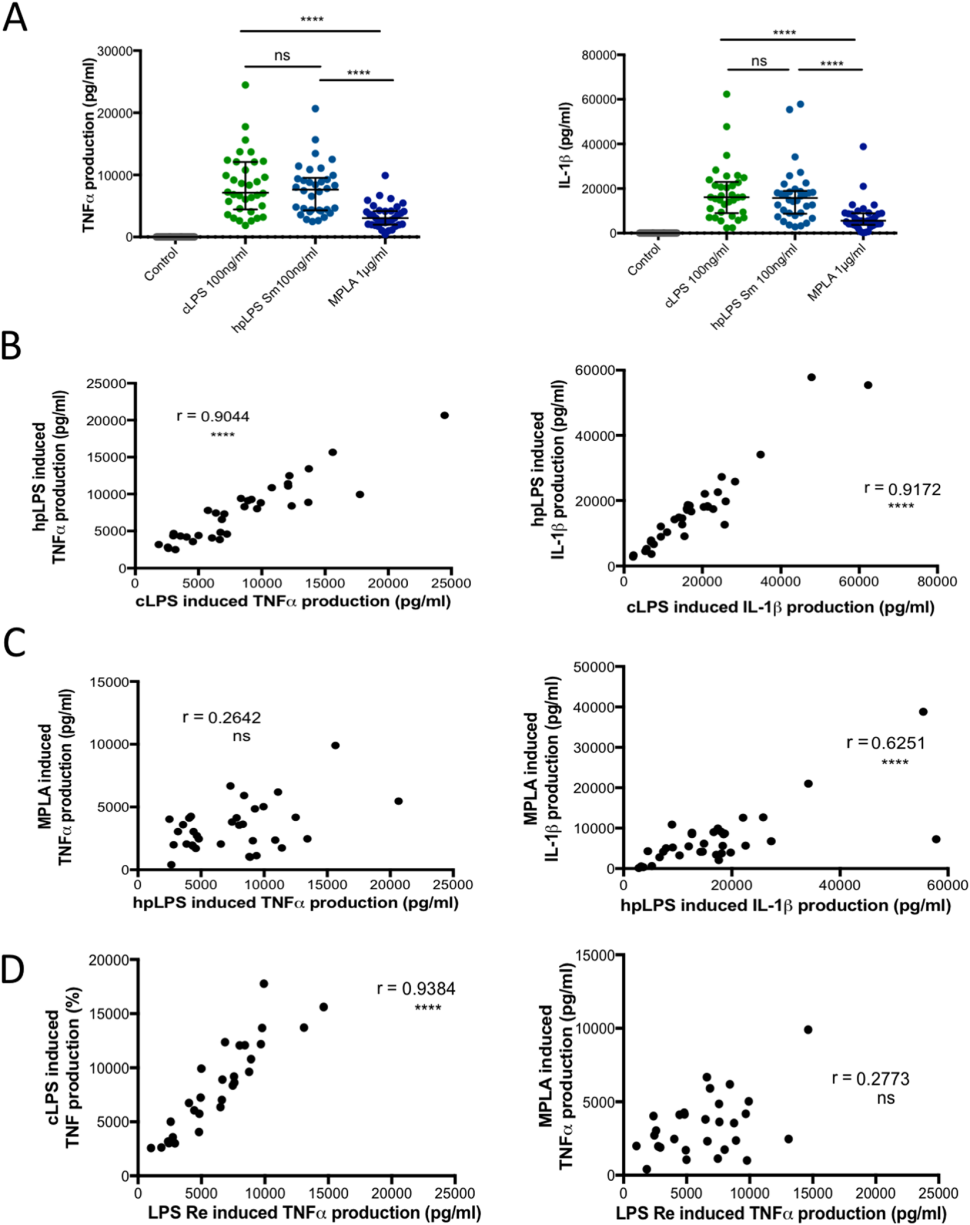
TNF and IL-1β production by human blood adherent mononuclear cells stimulated with 100 ng/ml conventional *Escherichia coli* smooth LPS O111:B4 (cLPS), highly purified smooth *Salmonella abortus equi* LPS (hpLPS), highly purified rough *S. minnesota* Re595 LPS (LPS Re) or 1 µg/ml MPLA. (**A**) Individual responsiveness of n = 34 individuals. (**B**) Correlations between individual responsiveness to cLPS and hpLPS in terms of TNF and IL-1β production. (**C**) Correlations between individual responsiveness to MPLA and hpLPS in terms of TNF and IL-1β production. (**D**) Correlations between individual responsiveness to rough Re LPS and cLPS or MPLA and in terms of TNF. Each dot represents one individual donor. ****p < 0.0001.

Among humans the intensity of the production of cytokines, such as TNF and IL-1β, in response to LPS^20,21^ varies greatly. This was confirmed in our own investigation (Fig. 1A). Preliminary experiments and multiple sources of published data, including ours^22,23^, have established that greater concentrations of Lipid A/MPLA are needed to achieve similar activation levels as seen when whole LPS molecule are used. In spite of using a ten-fold higher concentration of MPLA than what was used with conventional LPS or highly purified LPS, the production of TNF and IL-1β was significantly lower with MPLA introduction. A high and significant correlation was observed between the levels of TNF released by human monocytes from different donors in response to either conventional *Escherichia coli* LPS or highly purified *Salmonella abortus equi* LPS (r = 0.90; p < 0.0001). A similar high correlation between IL-1β and LPSs introduction was also observed (r = 0.92; p < 0.0001) (Fig. 1B). In contrast, no correlation was seen to exists between conventional *E. coli* LPS and MPLA (TNF: r = 0.29; data not shown) or between highly purified LPS and MPLA (TNF: r = 0.26; Fig. 1C). However, there was a significant correlation between hpLPS and MPLA when IL-1β was analyzed (r = 0.63, p < 0.0001). Similar observations were made when a highly purified rough Re LPS was used and compared to highly purified LPS (r = 0.94, p < 0.0001) or MPLA (r = 0.28, ns) in terms of TNF production (Fig. 1D) or IL-1β production (data not shown). Importantly, neither cLPS, hpLPS, nor MPLA, at the concentrations used, significantly affected the viability of the adherent cells compared to the controls (medium alone) as assessed by the MTT assay. This suggests that neither toxicity, nor pyroptosis was involved in the observation.

### Anti-inflammatory cytokines production

**Figure 2:**
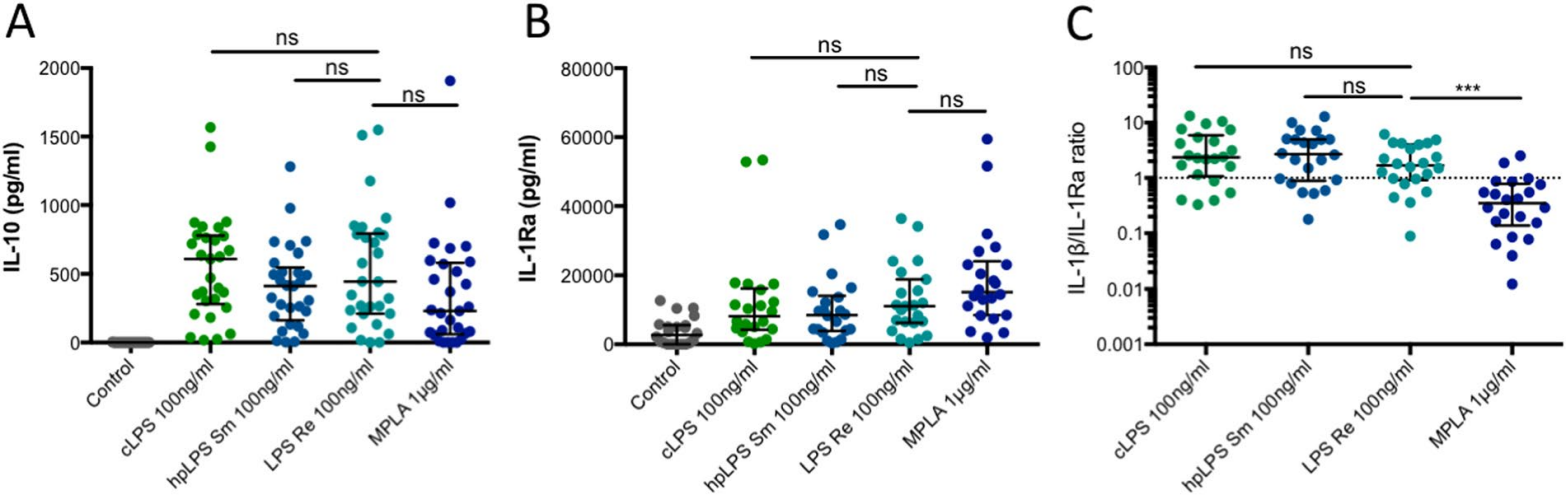
(**A**) IL-10 and (**B**) IL-1Ra production by human adherent mononuclear cells activated with either conventional smooth *E. coli* O111:B4 LPS (cLPS) (100 ng/ml), highly purified *S. abortus equi* LPS (hpLPS) (100 ng/ml), highly purified rough *S. minnesota* LPS (ReLPS) (100 ng/ml) or MPLA (1 µg/ml) for different donors. (**C**) Ratios between IL-1β and IL-1Ra for individual donors. Each dot represents one individual donor. ***p < 0.001.

As shown in Fig. 2, MPLA (1 µg/mL) induced the production of IL-10 and IL-1 receptor antagonist (IL-1Ra) within the range of the levels induced by conventional and highly purified LPSs (100 ng/mL). It is important to note that a release of IL-1Ra is observed in the absence of any stimulation. Again a great heterogeneity exists amongst the different donors. Interestingly, the ratio IL-1β/IL-1Ra was significantly lower (p < 0.001) in response to MPLA as compared to any of the LPSs.

### Monocyte activation by LPS and MPLA requires TLR4

**Figure 3:**
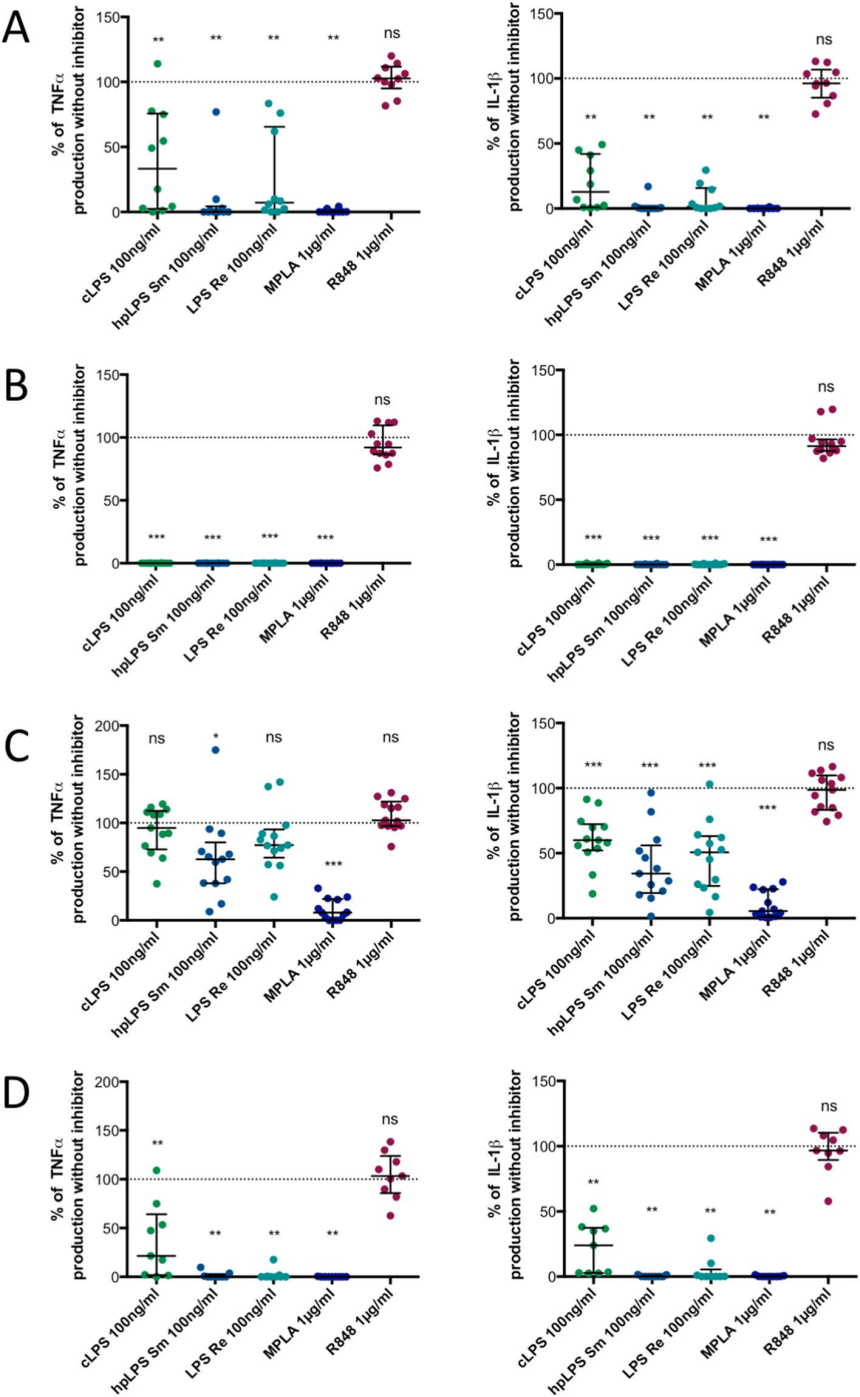
Both LPS and MPLA activate human adherent mononuclear cells via TLR4. (**A**) Inhibition by 1 µg/ml of *Rhodobacter sphaeroides* LPS of TNF and IL-1β release induced by either conventional smooth *E. coli* O111:B4 LPS (cLPS) (100 ng/ml), highly purified *S. abortus equi* LPS (hpLPS) (100 ng/ml), highly purified rough *S. minnesota* LPS (ReLPS) (100 ng/ml) or MPLA (1 µg/ml) for different donors. (**B**) Similar experiments performed with 10 µg/ml of *R. sphaeroides* LPS. (**C**) Similar inhibitory experiments performed with 10 nM or (**D**) 100 nM Eritoran (each dot represents one individual donor). Neither *Rhodobacter sphaeroides* LPS nor Eritoran affected the cytokine production in the absence of agonists (data not shown). Results are expressed as percent of the individual responses in the absence of inhibitors. *p ≤ 0.5; **p < 0.01; ***p < 0.001.

To decipher whether both LPS & MPLA activate human monocytes via TLR4 we used two different TLR4 antagonists: *Rhodobacter sphaeroides* LPS^24^ and Eritoran^25^. As shown in Fig. 3A–D, both antagonists prevented TNF and IL-1β production induced by LPS and MPLA while the cytokine production induced by a TLR7/8 ligand was unaffected. When used at the highest concentration (10 µg/mL) *R. sphaeroides* LPS fully abolished the production of TNF and IL-1β by all LPSs and MPLA (Fig. 3B). Similarly, the highest concentration (100 nM) of Eritoran completely abolished the cytokine production induced by highly purified LPSs, but production was observed in some donor samples when conventional *E. coli* LPS was used (Fig. 3D). These results illustrate that, similarly to murine macrophages^14^, MPLA acts via TLR4 in human monocytes.

### CD14 is dispensable for MPLA activation

**Figure 4:**
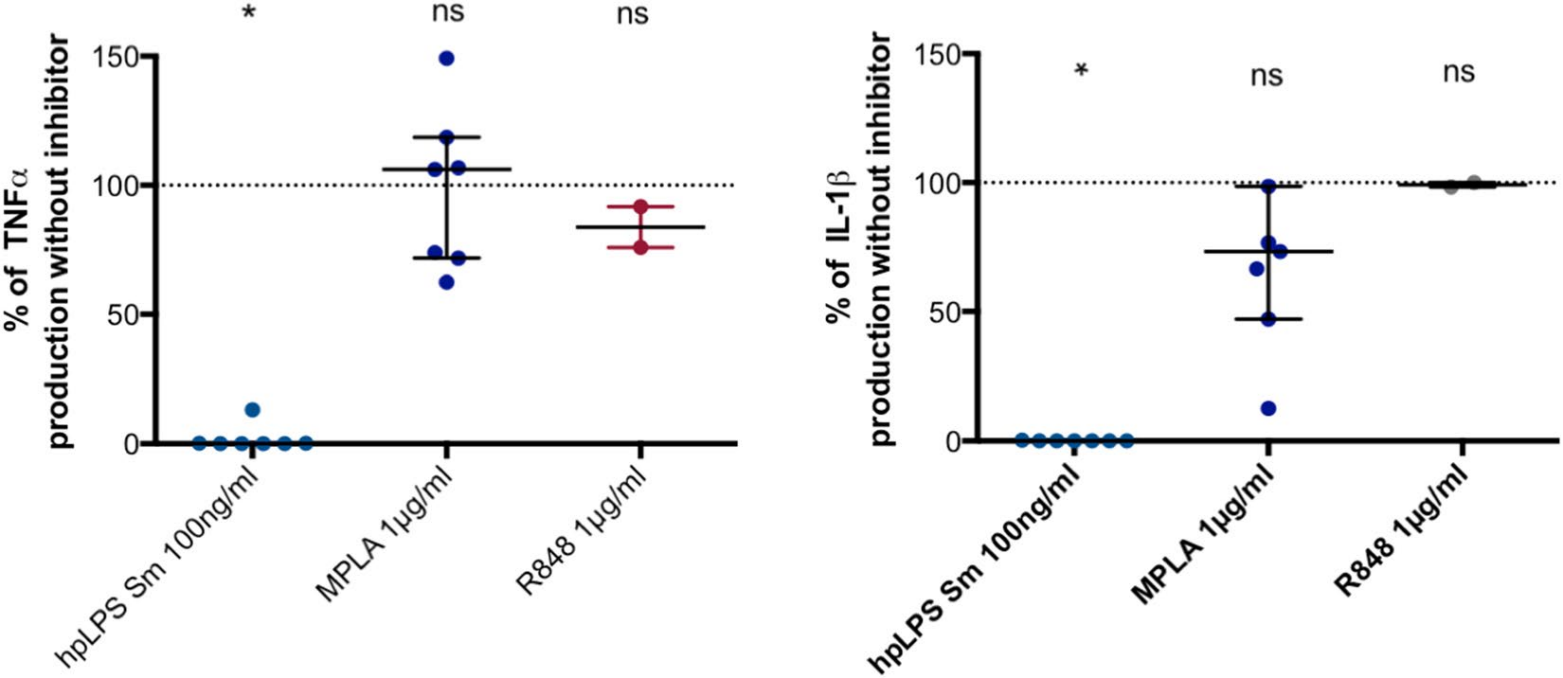
TNF and IL-1β production by highly purified *S. abortus equi* LPS (hpLPS) (100 ng/ml) or MPLA (1 µg/ml) or R848 (1 µg/ml) for different donors in the presence of anti-CD14 antibodies (2.5 µM). Anti-CD14 antibodies did not affect the cytokine production in the absence of agonists (data not shown). Each dot represents one individual donor. Results are expressed as percent of the individual responses in the absence of antibodies. *p < 0.05.

We investigated whether CD14 is a prerequisite for MPLA activation as it is for LPSs. As shown in Fig. 4, anti-CD14 antibodies fully inhibited hpLPS-induced TNF and IL-1β. In contrast, MPLA activated TNF production was minimally modified, and there was no significantly lower production of IL-1β.

### Similarities between MPLA and LPS induced IL-6

**Figure 5:**
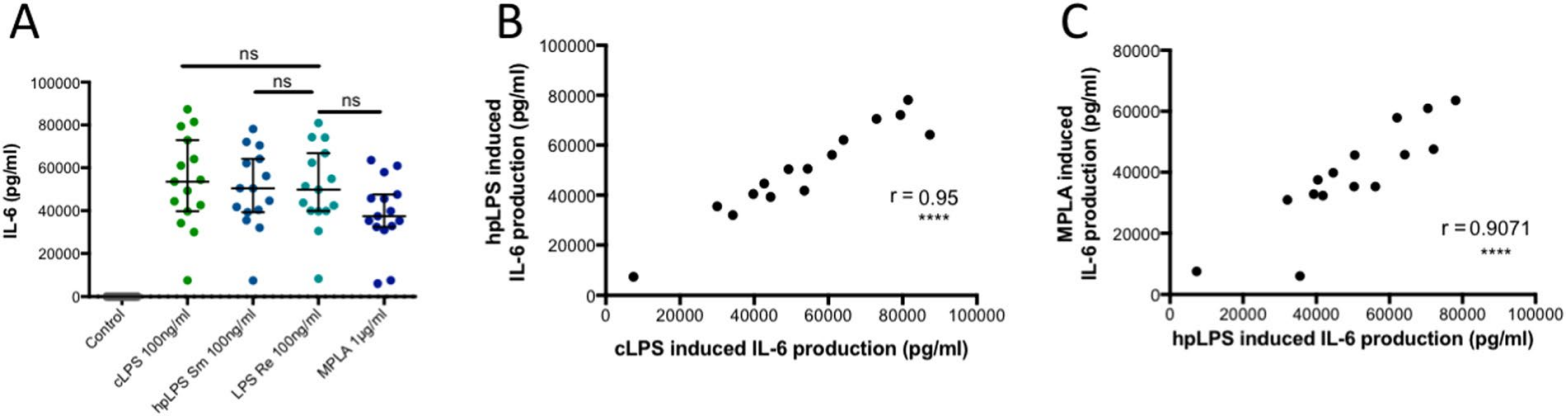
(**A**) IL-6 production by human adherent mononuclear cells activated by either conventional smooth *E. coli* O111:B4 LPS (cLPS) (100 ng/ml), highly purified S*. abortus equi* LPS (hpLPS) (100 ng/ml), highly purified rough *S. minnesota* LPS (ReLPS) (100 ng/ml) or MPLA (1 µg/ml) for different donors. (**B**) and (**C**) correlation for IL-6 production by adherent mononuclear cells from individual donors between, hpLPS and cLPS or MPLA. Each dot represents one individual donor. ****p < 0.0001.

The observations of the production of IL-6 induced by LPSs and MPLA were markedly different from those of TNF and IL-1β (Fig. 5). There was a remarkable correlation between the production of IL-6 induced by hpLPS and that induced by MPLA (r = 0.91; Fig. 5C). Samples from high producer donors of IL-6 that where identified after LPSs introduction were also identified as high producer when exposed to MPLA. Similar positive correlations were observed with MPLA, cLPS (r = 0.83), and Re LPS (r = 0.87) (data not shown). TLR4 inhibitors (*Rhodobacter sphaeroides* and eritoran) abolished the LPS and MPLA induced IL-6 production, while anti-CD14 antibodies only prevented LPS induced IL-6 production (data not shown). These results suggest that the cascade events, after the TLR4/MD2 activation, leading to IL-6 production are rather similar for both LPS and MPLA, and distinct from those leading to TNF production.

### Contribution of endocytosis in TNF production

**Figure 6:**
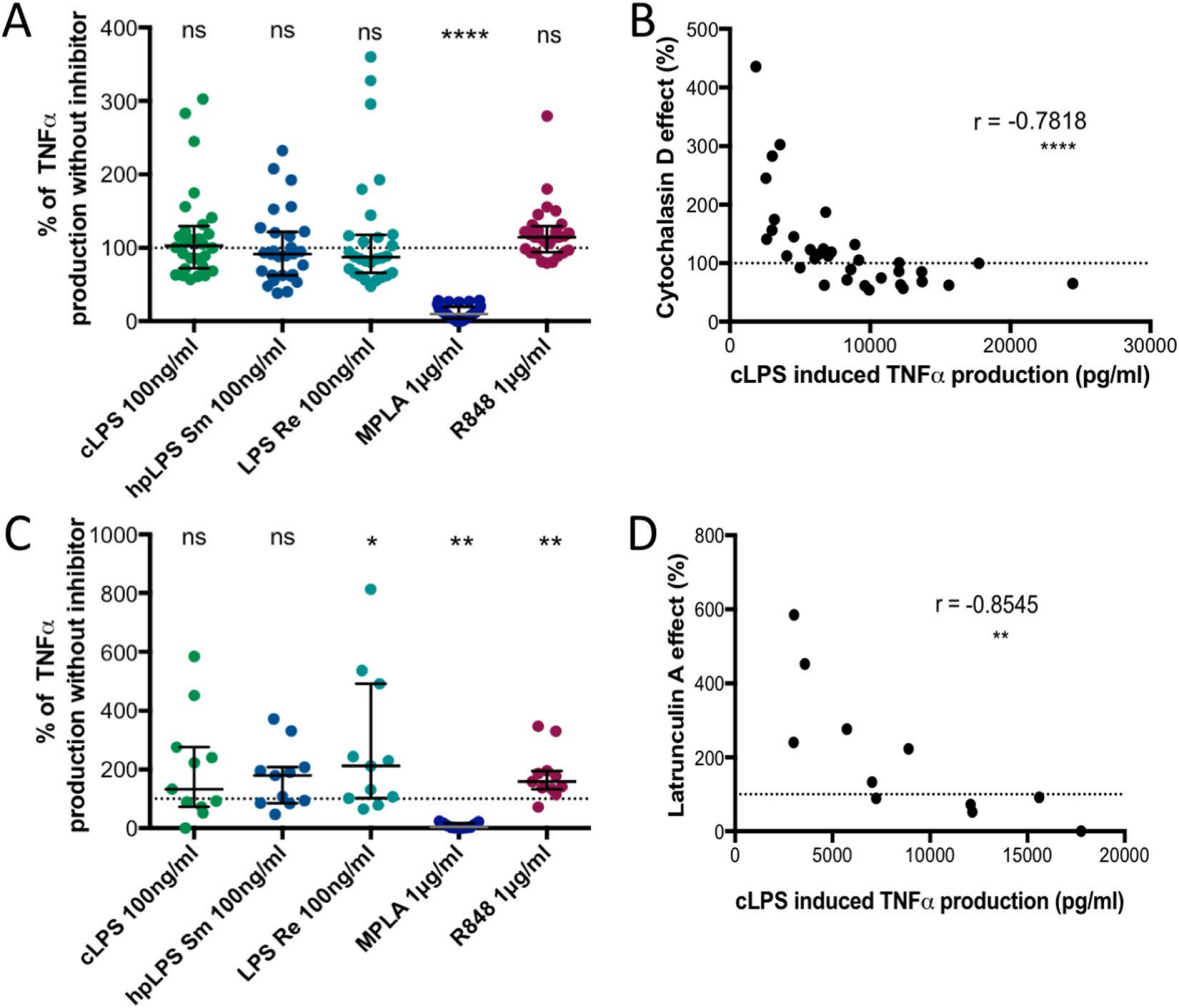
TNF production in the presence of actin polymerization inhibitors. (**A**) Inhibition by 3 µM cytochalasin D of TNF production by human adherent mononuclear cells activated by either conventional smooth *E. coli* O111:B4 LPS (cLPS) (100 ng/ml), highly purified *S. abortus equi* LPS (hpLPS) (100 ng/ml), highly purified rough *S. minnesota* LPS (ReLPS) (100 ng/ml) or MPLA (1 µg/ml) for different donors. (**B**) Correlation between the levels of TNF produced in response to conventional *E. coli* LPS in absence (horizontal axis) or in the presence (vertical axis) of cytochalasin D. (**C**) Similar experiments performed with 2 µM latrunculin A. (**D**) Correlation between the levels of TNF produced in response to conventional *E. coli* LPS in absence (horizontal axis) or in the presence (vertical axis) of latrunculin A. Neither cytochalasin D nor latrunculin A affected the cytokine production in the absence of agonists (data not shown). Each dot represents one individual donor. Results are expressed as percent of the individual responses in the absence of inhibitors (**A**,**C**) or as absolute levels of TNF (**B**,**D**). *p ≤ 0.5; **p < 0.01; ****p < 0.0001.

Cytochalasin D is an inhibitor of actin polymerization, and as such it is an inhibitor of phagocytosis^26^ and endocytosis^27^. As shown in Fig. 6A, the addition of cytochalasin D lead to either an increased or a decreased in the production of TNF depending upon the sample’s donors response to LPSs. Interestingly, it appears that the increased production was mainly observed among the samples from low TNF responders whereas the decreased production was mainly found among the samples from high TNF responders (r = −0.78, Fig. 6B). Similar observations were made with IL-1β (data not shown). In contrast, in samples from all donors, TNF and IL-1β (data not shown) productions in response to MPLA were essentially abolished by the addition of cytochalasin D (Fig. 6A). These results were reproduced when using another inhibitor, latrunculin A, which also prevents endocytosis^28^ (Fig. 6C,D). Similarly, latrunculin A lead to an enhanced release of TNF among the samples from low producers and an inhibited release of TNF among the samples from high producers in the presence of LPS (r = −0.85, Fig. 6D). These results demonstrate that the internalization of MPLA is a prerequisite for TNF induction in all cases, whereas for LPS, TNF induction is donor dependent with the effect of internalization inhibition being mainly observed among samples from high TNF producers.

### IL-1β similarly controls LPS and MPLA induced TNF production

**Figure 7:**
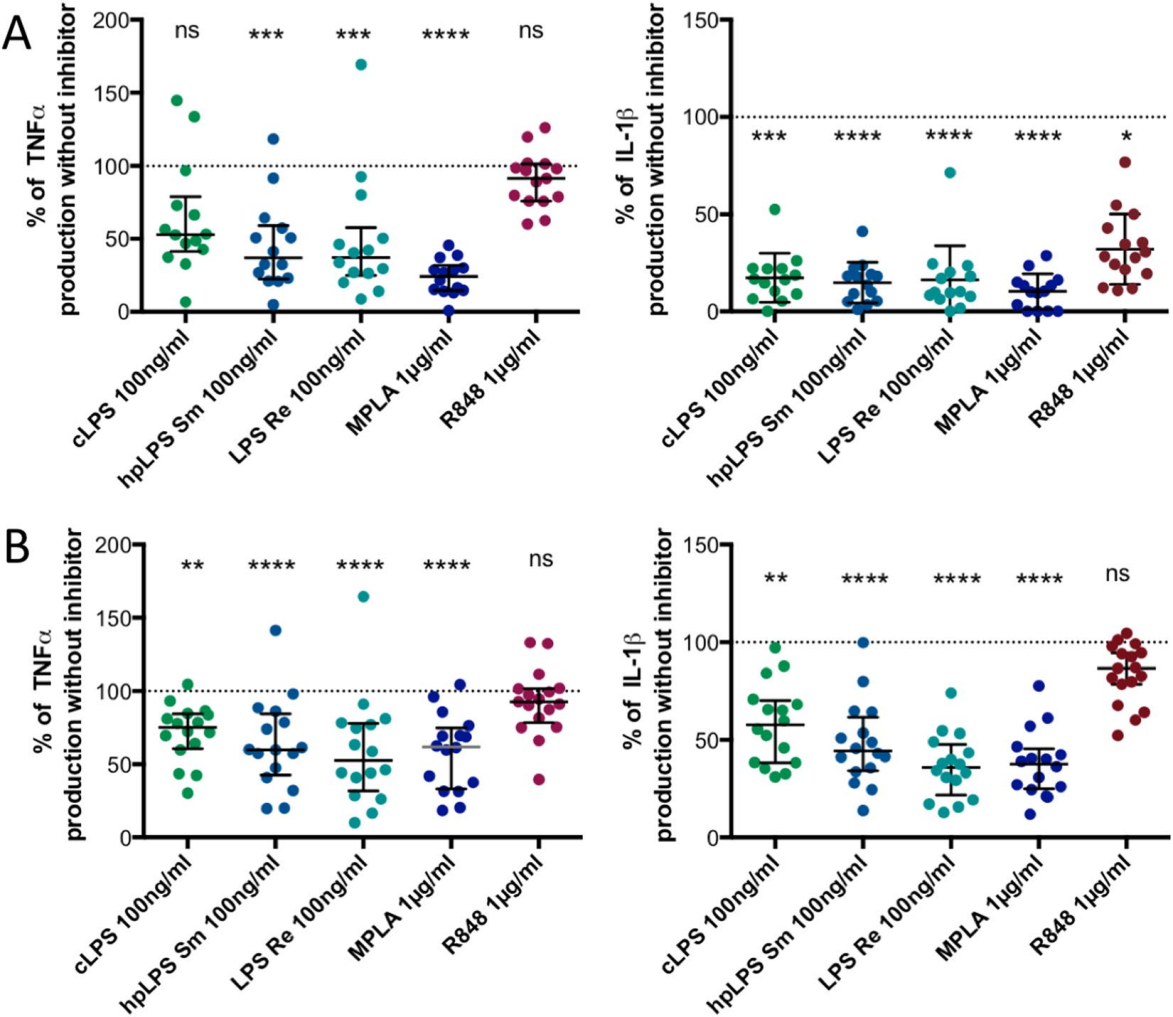
(**A**) Inhibition of TNF and IL-1β production by pan-caspase inhibitor (Z-VAD 20 µM) of human adherent mononuclear cells activated by either conventional smooth *E. coli* O111:B4 LPS (cLPS) (100 ng/ml), highly purified *S. abortus equi* LPS (hpLPS) (100 ng/ml), highly purified rough *S. minnesota* LPS (ReLPS) (100 ng/ml) or MPLA (1 µg/ml) for different donors. (**B**) Similar experiments performed in the presence of IL-1Ra (100 µg/ml). Each dot represents one individual donor. Neither Z-VAD nor IL-1Ra affected the cytokine production in the absence of agonists (data not shown). Results are expressed as percent of the individual responses in the absence of inhibitors. *p < 0.5; **p < 0.01; ***p < 0.001; ****p < 0.0001.

Since we have studied the supernatants of overnight cultures (20 h incubation), and since autocrine loops have been well-established^29^, we investigated whether the feedback effects of the released IL-1β could help discriminate between LPS and MPLA-induced TNF induction. As shown in Fig. 7A, when IL-1β production was prevented by the addition of a pan-caspase inhibitor, TNF production was reduced independently of the TLR4 activators. In contrast, TNF production induced by a TLR7/TLR8 remained unchanged. Similarly, when the autocrine loop of IL-1β was prevented by the addition of an excess of IL-1Ra the productions of TNF were significantly reduced (Fig. 7B). This was not, or less observed when a TLR7/8 agonist was employed.

### MPLA fails to induce cross-tolerance to cLPS and a TLR2 ligand

**Figure 8:**
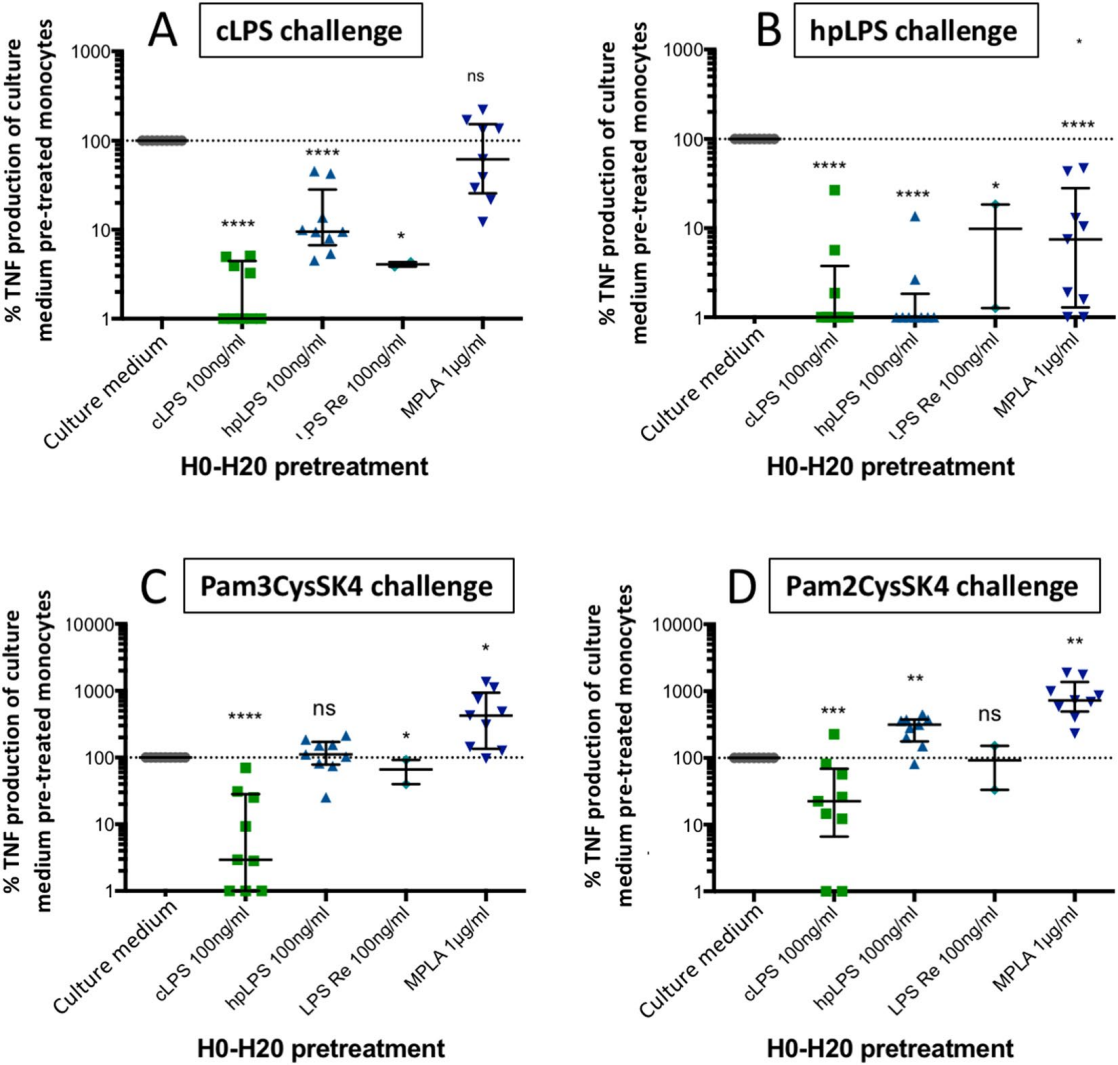
Induction of homo- and cross-tolerance. Human adherent mononuclear cells were pretreated for 20 h with either conventional smooth *E. coli* O111:B4 LPS (cLPS) (100 ng/ml), highly purified *S. abortus equi* LPS (hpLPS) (100 ng/ml), highly purified rough *S. minnesota* R595 LPS (ReLPS) (100 ng/ml) or MPLA (1 µg/ml). After elimination of the supernatants and washings, cells were re-challenge with either cLPS (**A**), hpLPS (**B**), Pam_3_CysSK_4_ (**C**) or Pam_2_CysSK_4_ (**D**), and TNF production was assessed 20 h later. The results are expressed as percent of the production of individual donors to the different agonists used for challenge in cells, which had not been pre-treated with TLR agonists. Each dot represents individual donors. *p ≤ 0.5; **p < 0.01; ***p < 0.001; ****p < 0.0001.

Endotoxin tolerance is a well-known phenomenon that prevents cells from being responsive to a second challenge with LPS^30,31^. There is a cross-tolerance between the TLR4 and TLR2 ligands^32,33^. We showed that conventional LPS tolerized monocytes to itself and to highly purified LPS (Fig. 8A,B). In contrast, highly purified LPS, which was able to tolerize monocytes to itself, was less efficient at tolerizing monocytes to conventional LPS. Conventional LPS, but not highly purified LPS crosstolerized cells to Pam_3_CysSK_4_, a TLR1/TLR2 ligand (Fig. 8C,D). MPLA induced homotolerance (data not shown) and tolerance to highly purified LPS, but did not lead to tolerization to conventional LPS (Fig. 8A,B). Furthermore, not only did MPLAs fail to induce cross-tolerance to TLR1/TLR2 (Pam3CysSK4) and TLR2/TLR6 (Pam2CysSK4) ligands, it even primed the cellular response to them (Fig. 8C,D).

### Signaling pathways involved in response to LPS and MPLA

**Figure 9:**
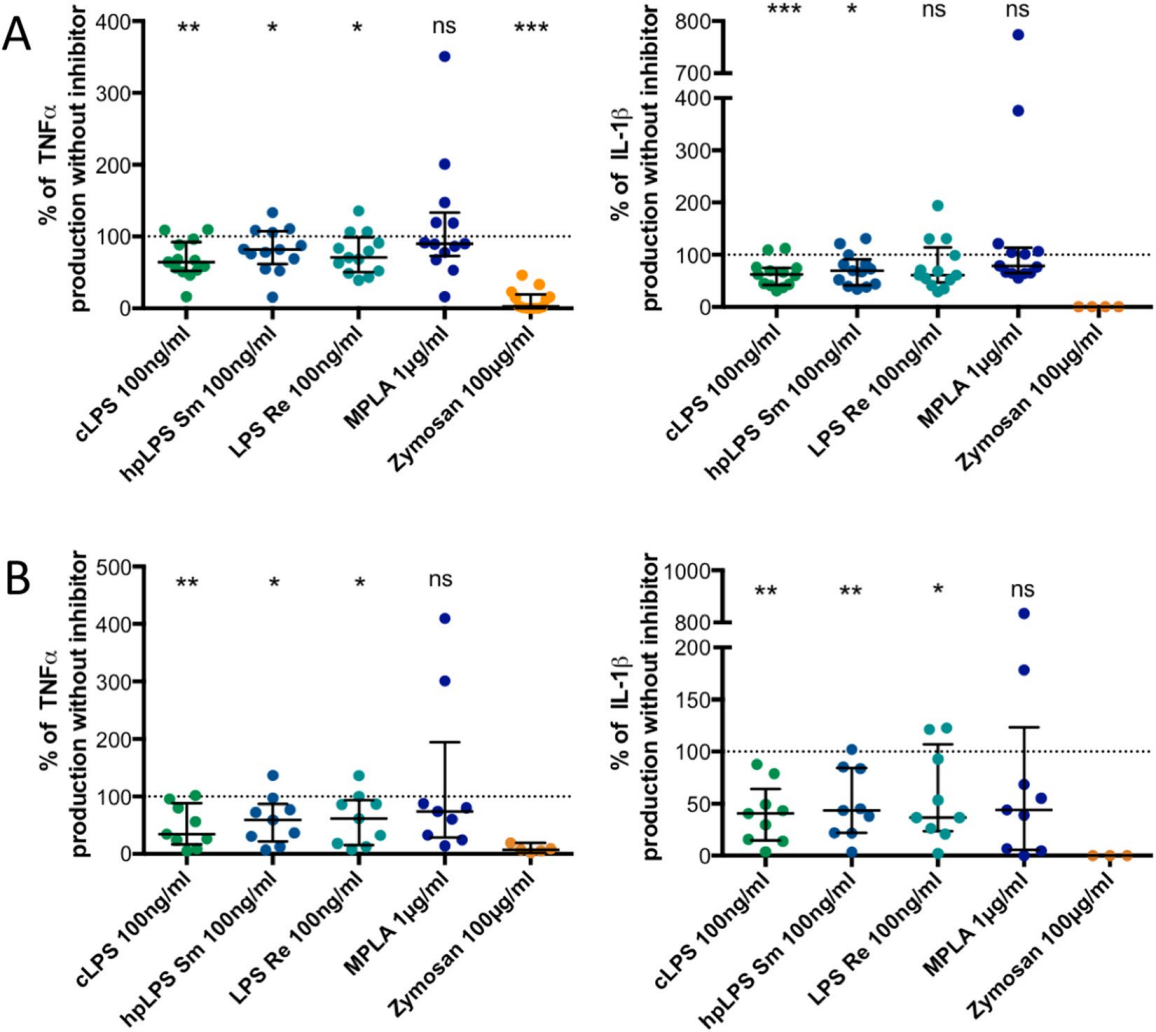
Effect of Syk inhibitor R406 (**A**) 1 µM, or (**B**) 5 µM on TNF and IL-1β production by human adherent mononuclear cells activated by either conventional smooth *E. coli* O111:B4 LPS (cLPS) (100 ng/ml), highly purified *S. abortus equi* LPS (hpLPS) (100 ng/ml), highly purified rough *S. minnesota* LPS (ReLPS) (100 ng/ml), MPLA (1 µg/ml) or zymosan depleted (100 µg/ml) for different donors. R406 did not affect the cytokine production in the absence of agonists (data not shown). Each dot represents one individual donor. Results are expressed as percent of the individual responses in the absence of inhibitor. *p ≤ 0.5; **p < 0.01; ****p < 0.001; ***p < 0.0001.

The Syk pathway has been recently demonstrated to be associated with a LPS-co-receptor (namely CD300b)^13^. We investigated whether a Syk inhibitor (compound R406) could help discriminate between LPS and MPLA induced activation (Fig. 9).

TNF and IL-1β productions induced by all the LPSs were altered by the addition of the Syk inhibitor. As expected, the compound R406 fully prevented cytokine productions induced by zymosan, but it did not have a clear effect on MPLA induced TNF and IL-1β production.

Various Western blot analyses were carried out with the aim to differentiate the activation of human monocytes via LPS *versus* MPLA. Of note, we failed to find any significant differences in ERK1/2, p-38 MAP kinase, JNK, and IκBα phosphorylation (data not shown).

## Discussion

Our investigation confirms the huge cytokine production heterogeneity seen between donors in response to LPS^20,21^. The different levels of TNF and IL-1β (observed in samples from high, intermediate, or low producers) are similarly reached by using conventional LPS (containing TLR2 agonist contaminants)^34^, highly purified LPS, and smooth or rough endotoxin. This was illustrated by the strong correlations observed between these different agonists. As expected, the different enterobacteriaceae LPSs behaved similarly independently of their bacterial origin. This reflects that *Salmonella* LPS is a mixture of differently acylated lipid A, including large amounts of hexa-acylated ones similar to that of *E. coli*^35^. It is important to note that in our study we did not address the influence of gender that has also been shown to influence the intensity of the response^36^. Surprisingly, the effect of cytochalasin D and latrunculin A, which prevent the internalization of LPS, had different consequences when added to human adherent mononuclear cells. In samples from low producers it enhanced the production of TNF while in samples from high producers it decreased the production. This observation suggests that low producers have their cells mainly activated from the cell surface. Blocking internalization could offer up more ligands to the LPS surface receptor ending with an increased signal. In contrast, in high producers the signal could be delivered from both the cell surface and after internalization. Preventing internalization could have withdrawn an additional intracellular pathway accounting for the lower production amongst the high producers.

As previously shown with the lipid A molecules^22,23,37,38^, MPLA is a weak inducer of TNF and IL-1β when compared to LPS. Using a tenfold higher concentration of MPLA than LPS still ended with lower TNF and IL-1β production in human monocytes than when stimulated with LPS. This aligns with a study performed with monocyte-derived dendritic cells, which were poor producers of IL-12 when activated with MPLA when compared to LPS^38,39^. In agreement with many other studies, we showed that both LPS and MPLA need TLR4 to activate the cells^14,36,40^. Two TLR4 antagonists (*R. sphaeroides* LPS and Eritoran) display a significant inhibitory capacity for the cytokine production induced by LPS or MPLA. Nevertheless, there was no correlation between the levels of TNF obtained upon LPS activation or with MPLA activation amongst the samples from different donors. This strongly suggests that the signaling cascade ending with TNF release in response to LPS and MPLA, downstream of TLR4, are not similar. In contrast, when IL-6 was measured there was a strong correlation, amongst the samples from all the different donors, between the levels of this cytokine in the presence of cLPS, hpLPS, and MPLA (0.83 < r < 0.91). Accordingly, in contrast to TNF induction, the signaling cascade leading to IL-6 production induced by LPS and MPLA could be similar. The levels of IL-6 obtained with 100 ng/mL LPS and 1 µg/mL MPLA were similar. The relative high capacity of MPLA to induce IL-6 and its low capacity to induce TNF and IL-1β could contribute to its adjuvanticity. IL-6 is a potent B-lymphocyte activator^41^ with limited toxicity. Another observed similarity between LPS and MPLA was their relative dependency of TNF production on the production of IL-1β. This similarity was seen when both caspase 1 inhibited IL-1β production and excess IL-1Ra inhibited IL-1β production lead to a reduction of TNF production for both activators. We also observed, in agreement with a previous report, the potent ability of MPLA to induced IL-10 and IL-1Ra^42^. The lower ratio IL-1β/IL-1Ra obtained with MPLA when compared to LPS may partially explain the reduced toxicity of the molecule. Interestingly, Luan *et al*.^43^ recently reported that MPLA and LPS induce a similar transcriptome profile in human blood, although LPS and MPLA differentially regulated 136 and 130 genes respectively. It is important to keep in mind that this type of gene analysis does represent the relative amount of protein expressed in cells.

In contrast to LPS, MPLA activation was shown to be CD14 independent. Anti-CD14 antibodies did not modify the cytokine production in response to MPLA while they did prevent it when LPSs were employed. This is in agreement with reported experiments using bone marrow derived dendritic cells from CD14 deficient mice^44^ and with experiments performed with human dendritic cells^45^. These observations fit with the fact that the KDO molecules are required for the binding of LPS to CD14. This explains the binding of Re LPS to CD14, but it does not suggest any binding of lipid A to CD14^46^. Of note, internalization of LPS was shown to be CD14 dependent^47^. This is clearly not the case for MPLA.

Another striking difference between LPS and MPLA was the failure of MPLA to induce tolerance to cLPS, which contain TLR2 agonist contaminants, while inducing tolerance to hpLPS, which is a strict TLR4 agonist. A similar cross-tolerance between LPS and MPLA was already reported with human monocytes in a study looking at superoxide and hydrogen peroxide production^48^. In the experiments performed *in vivo* MPLA was also shown to provide tolerance against lethal dose of LPS. Although, concentrations of greater than 20,000 times the concentration of LPS for MPLA were needed to achieve a similar protective effect^49^. The *in vivo* tolerance induced by MPLA was accompanied by a reduction of colony stimulating activity, a reduction of interferon activity, and a reduction of TNF activity in the sera of LPS-injected mice^50^. Interestingly, cLPS could tolerize human adherent mononuclear cells to TLR2 ligands while hpLPS was not able to tolerize, and MPLA primed the response to TLR1/TLR2 or TLR2/TLR6 ligands. This priming effect of MPLA is reminiscent of the priming effect of TLR3 agonists on the subsequent activation by TLR2 and TLR4 agonists^51^. This observation further illustrates the difference in the signaling pathways between LPS and MPLA ending to TNF production.

An additional major distinction between the cytokine induction caused by LPS and MPLA was observed when cultures were performed in the presence of cytochalasin D or latrunculin A. For samples from all donors these drugs significantly reduced MPLA induced TNF and IL-1β productions. While the responses for LPS induced TNF and IL-1β production, under both drugs, were donor dependent. These results suggest that the activating signals, following the interaction between MPLA and the human adherent mononuclear cells, are linked to an intracellular process. This is in contrast with numerous investigations, which reported only limited activation when LPS or lipid A were delivered intracellularly by liposome encapsulation^52–54^. However, it is possible that the delivery of the LPS molecules after internalization of liposomes differs from the one that occurs after their interaction with the cell membrane. In mice, MPLA has been described to favor the TRIF pathway, which is induced after the internalization of the molecule^14,55^. More investigations are needed to demonstrate that this is also the case with human mononuclear adherent cells. It also remains to be deciphered whether surface TLR4/MD2 captures MPLA and contributes to its internalization before inducing the signaling pathway, or whether MPLA is internalized following a lipid - lipid interaction and activates intracellular TLR4 that are present within human monocytes^56^. Regarding the intracellular signaling cascade we failed to find clear cut differences between LPS and MPLA induced phosphorylation changes of p38 and ERK1/2 (in agreement with previous investigations^57^), or of JNK^40^ and IκBα.

Most interestingly, our investigations clearly illustrate the divergence between data acquired with murine cells and those obtained with human blood leukocytes, in agreement with the previous observation establishing that LPS triggers IL-1β secretion in human, but not in murine monocytes^58^. The main divergence was the capacity of MPLA to induce significant amounts of IL-1β release by activated human blood adherent mononuclear cells while murine macrophages do not produce this cytokine in response to MPLA^59^. The absence of IL-1β production within murine cells was due to a failure of MPLA to activate caspase 1^55^ and a consequence of its TRIF-biased signaling^60^. Of note, our observation with primary human cells is in agreement with a previous study that used whole blood^43^, but is different from another previously reported study that used a human macrophage-like cell line (THP-1) that failed to observe the release of IL-1β^55^.

Altogether, using freshly isolated human blood mononuclear cells we have shown the differences in events ending with either TNF or IL-6 production in response to LPS or MPLA introduction. In addition to the well-known low capacity of MPLA to activate human monocytes^60^, we have observed specific properties of MPLA that distinguishes it from the LPS induced cytokines. Particularly, we noticed the absolute prerequisite of MPLA internalization for TNF induction. We have shown a new property of MPLA in priming human adherent mononuclear cells to a TLR2 ligand, an event that distinguishes it from LPS. Finally, we have identified a major difference between the responsiveness of murine and human cells to MPLA in terms of IL-1β induction.
